# Triple Perovskite Nd_1.5_Ba_1.5_CoFeMnO_9−δ_-Sm_0.2_Ce_0.8_O_1.9_ Composite as Cathodes for the Intermediate Temperature Solid Oxide Fuel Cells

**DOI:** 10.3390/ma15103663

**Published:** 2022-05-20

**Authors:** Yunru Chen, Tao Yu, Jiang Jin, Hua Zhang

**Affiliations:** College of Materials Science and Engineering, Nanjing Tech University, Nanjing 211816, China; chenyruuu@163.com (Y.C.); 201961103046@njtech.edu.cn (T.Y.); jinjiang@njtech.edu.cn (J.J.)

**Keywords:** triple perovskite structure, composite cathode, electrochemical performance, thermal expansion

## Abstract

Triple perovskite has been recently developed for the intermediate temperature solid oxide fuel cell (IT-SOFC). The performance of Nd_1.5_Ba_1.5_CoFeMnO_9−δ_ (NBCFM) cathodes for IT-SOFC is investigated in this work. The interfacial polarization resistance (R_P_) of NBCFM is 1.1273 Ω cm^2^~0.1587 Ω cm^2^ in the range of 700–800 °C, showing good electrochemical performance. The linear thermal expansion coefficient of NBCFM is 17.40 × 10^−6^ K^−1^ at 40–800 °C, which is significantly higher than that of the electrolyte. In order to further improve the electrochemical performance and reduce the thermal expansion coefficient (TEC) of NBCFM, Ce_0.8_Sm_0.2_O_2−δ_ (SDC) is mixed with NBCFM to prepare an NBCFM-*x*SDC composite cathode (*x* = 0, 10, 20, 30, 40 wt.%). The thermal expansion coefficient decreases monotonically from 17.40 × 10^−6^ K^−1^ to 15.25 × 10^−6^ K^−1^. The surface oxygen exchange coefficient of NBCFM-*x*SDC at a given temperature increases from 10^−4^ to 10^−3^ cm s^−1^ over the $p_{o_{2}}$ range from 0.01 to 0.09 atm, exhibiting fast surface exchange kinetics. The area specific resistance (ASR) of NBCFM-30%SDC is 0.06575 Ω cm^2^ at 800 °C, which is only 41% of the ASR value of NBCFM (0.15872 Ω cm^2^). The outstanding performance indicates the feasibility of NBCFM-30% SDC as an IT-SOFC cathode material. This study provides a promising strategy for designing high-performance composite cathodes for SOFCs based on triple perovskite structures.

## 1. Introduction

The solid oxide fuel cell (SOFC) has attracted much attention for its low pollutant emission and fuel flexibility in an attempt to effectively solve the energy crisis and environmental problems [1,2,3,4]. However, the high operating temperature has restricted the choice of available materials and affects the chemical stability and durability of cell components. In the past decade, developing IT-SOFCs with high power density and long-term stability has become one of the main research directions of SOFC [5,6]. One of the critical challenges is to develop cell components with low ohmic and polarization losses to reduce the cell resistivity and enhance the electrochemical performance at relative lower temperature [7,8,9]. It is recognized that polarization losses of the cell mainly result from the cathode. The cathode material should have high oxygen transport capacity, high electro-catalytic activity, and good compatibility with the electrolyte.

Recently, a family of oxides such as AA’Co_2_O_5+δ_ (A = RE and A’ = Ba, Sr) with layered or double perovskite-type structures has received widespread attention for its potential application in IT-SOFC cathodes [10]. In the A-site Ln^3+^ and Ba^2+^ cation-ordered LnBaCo_2_O_5+δ_, oxygen atoms can be partially or entirely removed from the lanthanide planes, significantly enhancing the diffusivity of oxide ions in the bulk and possibly supplying surface defect sites [11]. This family of oxides shows excellent ion transport properties and surface exchange kinetics and high electrical conductivity [12,13,14,15,16,17,18].

However, the thermal expansion coefficient (TEC) of LnBaCo_2_O_5+δ_ is too high to match with that of YSZ ((Y_2_O_3_)_0.08_-(ZrO_2_)_0.92_) or CeO_2_-based electrolytes (10~12 × 10^−6^ K^−1^) [19]. The mismatch between the cathode and electrolyte may trigger the cracking and delamination of cathode in the thermal cycling process, which is detrimental to the properties of SOFCs. The high TEC of LnBaCo_2_O_5+δ_ is related to the change of the electron-filling state of Co^3+^. This drawback can be minimized by substituting of the Co with transition-metal ions such as Fe, Mn, or Ni [20,21,22]. In addition, electrolyte materials as the second phase are often introduced to form composite cathodes, which can effectively reduce the TEC of the electrode, enlarge the electrochemically active area, and improve the electrode performance. For example, the area specific resistance (ASR) of LaBaCo_2_O_5+δ_-50%SDC was 0.052 Ω cm^2^ at 700 °C, which was less than the ASR of LBCO (0.3 Ω cm^2^ at 700 °C) [23]. The polarization resistance (R_P_) of SmBaCoCuO_5+δ_-50%GDC cathode was about seven times smaller than that of SmBaCoCuO_5+δ_ at 750 °C [24]. The microtubular solid oxide fuel cells with a 50vol%NdSrCo_2_O_5+δ_-50vol%GDC composite cathode presented good electrochemical performances. The cell R_P_ was 0.03 Ω cm^2^ at 800 °C, and the maximum power density was 0.67 W cm^−2^ [25].

The introduction of different cations and the ordering of the cations in the octahedral sites of the layered perovskite lead to a complex triple perovskite (A_2_A′B_2_B′O_9_) [26,27,28]. Nam-In Kim [29] studied Nd_1.5_Ba_1.5_CoFeMnO_9−δ_ (NBCFM) as a highly active and durable oxygen electrode catalyst for the oxygen evolution reaction (OER) and the oxygen reduction reaction (ORR). The NBCFM structure is generated by stacking three single perovskites. The nonstoichiometry δ value for NBCFM is 0.495, calculated from the cobalt oxidation state by using the iodometric titration method. These characteristics thus inspired us to extend our work to the practical application of NBCFM for an SOFC cathode to promote the development of SOFC.

Herein, Nd_1.5_Ba_1.5_CoFeMnO_9−δ_ (NBCFM) was synthesized by the glycine-nitrate process. The structure, thermal expansion behavior, and electrochemical performances of NBCFM were characterized. In order to overcome the thermal mismatching between the electrode and electrolyte and improve the electrochemical performance, SDC was added to NBCFM to prepare NBCFM-*x*SDC (*x* = 10, 20, 30 and 40 wt.%) composite cathodes. The structure, chemical compatibility, electrical conductivity, TEC, and oxygen surface exchange of these composite materials were investigated, and the electrochemical performance of NBCFM-*x*SDC as cathodes for IT-SOFC applications was evaluated.

## 2. Materials and Methods

### 2.1. Cathode Powder Synthesis

NBCFM powder was prepared by the glycine-nitrate process (GNP). A certain amount of metal nitrate precursors, Nd(NO_3_)_3_·6H_2_O (Aladdin Reagent Co., Ltd., AR., Shanghai, China), Ba(NO_3_)_2_ (China National Pharmaceutical Group Co., AR., Shenzhen, China), Co(NO_3_)_3_·6H_2_O (Aladdin Reagent Co., Ltd., AR. Shanghai, China), Fe(NO_3_)_3_·9H_2_O (Aladdin Reagent Co., Ltd., AR. Shanghai, China), Mn (NO_3_)_3_ (China National Pharmaceutical Group Co., AR. Shenzhen, China), and glycine (China National Pharmaceutical Group Co., AR. Shenzhen, China), were solubilized in deionized water. The molar ratio of metal ions to glycine was 1:2.5. The mixture solution was stirred and heated at 80 °C continuously until a homogeneous gel was formed. Then, the gel was heated continuously and ignited spontaneously. The precursor powder was calcined at 1200 °C for 2 h in the air. Ce_0.8_Sm_0.2_O_1.9_ (SDC) powder was also prepared by GNP with a molar ratio of metal ions to glycine of 1:1.8. The precursor powder was calcined at 700 °C for 2 h. The NBCFM-*x*SDC (*x* = 10, 20, 30 and 40 wt.%) powders were prepared by mixing SDC and NBCFM and grinding thoroughly for 2 h. The samples were denoted as NBCFM-10%SDC, NBCFM-20%SDC, NBCFM-30%SDC, and NBCFM-40%SDC, respectively.

### 2.2. Cell Fabrication

SDC electrolyte was prepared by pressing 0.6 g SDC powder into a pellet at 220 MPa. Then, the green pellet (φ = 10 mm) was calcined at 1400 °C for 6 h in air. The cathode slurry containing the prepared NBCFM-*x*SDC powder, terpineol, and ethyl cellulose was brushed on both sides of the SDC electrolyte and calcined at 1050 °C for 2 h. The final surface area of the symmetrical cell with the configuration of cathode/SDC/cathode was about 0.56 cm^2^. The half-cell with Ni-YSZ/YSZ/GDC was fabricated by the tape casting method, and the NBCFM-*x*SDC slurry was deposited onto the electrolyte film by a brush painting method, followed by firing at 1050 °C for 2 h. The anode-supported single cell with an active area of 0.28 cm^2^ for current-voltage (I-V) tests had the configuration of Ni-YSZ/YSZ/GDC/NBCFM-*x*SDC.

### 2.3. Characterization and Electrochemical Performance Test

The crystal structure was determined by X-ray diffraction (XRD, Rigaku Smartlab, Irvine, CA, USA) on Cu Kα radiation (λ = 1.5148 Å) with a step width of 0.02° and a scan range of 20°–80° at room temperature. The morphology of the sample and the interface contact between cathode and electrolyte were observed by a scanning electron microscope (SEM, ZEISS Sigma 300, Kino, Germany).

NBCFM and NBCFM-*x*SDC cathode powder were pressed into rods (64 mm × 3 mm × 5 mm) at the pressure of 100 MPa and sintered at 1150 °C for the measurement of the electrical conductivity and thermal expansion coefficient. The electrical conductivity was tested by a standard direct current (DC) four-terminal probe method in the temperature range of 450–800 °C in air. The oxygen diffusion behavior of NBCFM-*x*SDC was determined by the electrical conductivity relaxation (ECR) technique. When the oxygen pressure changed momentarily at a certain temperature, the corresponding conductivity of the material would also change to a new equilibrium. The oxygen partial pressure changed from 0.1 to 0.9 atm, and the flowing gas was kept at 50 mL min^−1^ for the ECR measurement. The data were recorded every three seconds and estimated by the least-squares model. TEC was measured in air from 40 to 800 °C by using a high-temperature thermal dilatometer (DIL 402PC NETZSCH, Selb, Germany). The electrochemical impedance spectroscopy was performed using an electrochemical analyzer (PARSTAT 227, AMETEK, Berwyn, IL, USA) in the temperature range of 500 °C–800 °C. Platinum wires were used to collect the current. The frequency range was 0.1 Hz–100 kHz, and the amplitude of the AC signal was 20 mV. The single cell performance was evaluated with hydrogen (97% H_2_/3% H_2_O) as fuel and ambient air as oxidant in 650–800 °C by an electrochemical workstation (RTS-8, Tianjin Nuoxinda Technology Co., Ltd., Tianjin, China).

## 3. Result and Discussion

### 3.1. Crystal Structure and Cathode Performance of NBCFM

**Figure 1:**
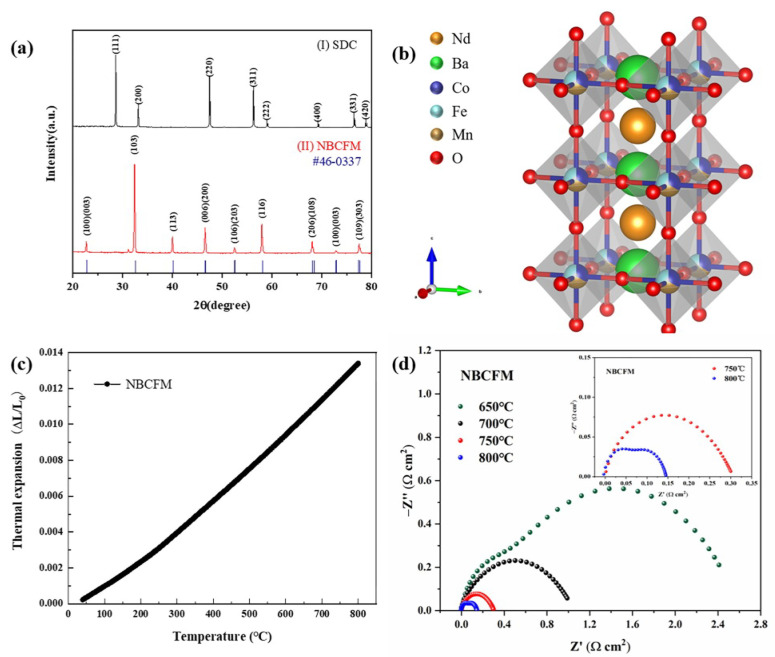
(**a**) X-ray patterns of SDC and NBCFM; (**b**) NBCFM crystal structure, (**c**) thermal expansion curves, and (**d**) impedance spectra at 650–800 °C of NBCFM; inset is the enlarged impedance spectra at 750 °C and 800 °C.

NBCFM structure was determined by the X-ray powder diffraction technique. The XRD patterns of NBCFM and SDC can be indexed as perovskite and fluorite structures with a space group of P4/mmm and Fm–3m, respectively (Figure 1a). The visualized crystal structure of the triple perovskite NBCFM is shown in Figure 1b. The lattice parameters of NBCFM and SDC are listed in Table 1.

The thermal expansion coefficient is an essential parameter for SOFC components. The mismatch of TEC between cathode and electrolyte can induce obvious strain during thermal cycling and lead to the cracking and delamination of the components. Figure 1c shows the thermal expansion curves of NBCFM cathode. The TEC value of NBCFM is 17.40 × 10^−6^ K^−1^, which is different from the TEC values of common electrolytes for IT-SOFC.

The electrochemical properties of NBCFM are studied by Electrochemical Impedance Spectroscopy (EIS). The EIS results of NBCFM in the temperature range of 650–800 °C are shown in Figure 1d. To clearly display the electrode polarization response, the ohmic response of the electrolyte and wire is returned to zero. The difference between intercepts of the high-frequency arc and low-frequency arc with the real axis corresponds to the interfacial polarization resistance (R_P_), which reflects the rate of oxygen reduction reaction (ORR). The R_P_ values of NBCFM are 0.1587 Ω cm^2^, 0.7239 Ω cm^2^, and 1.1273 Ω cm^2^ at 800 °C, 750 °C, and 700 °C, respectively. With the increase of temperature, the R_P_ of NBCFM decreases and the oxygen reduction reaction accelerates. At 800 °C, NBCFM exhibits low polarization loss, which can be attributed to its oxygen vacancy-rich structure. At high temperature, the increase of oxygen vacancy concentration is beneficial for the oxygen ion transport. This leads to the reduction of the polarization loss.

According to the above study, triple perovskite NBCFM exhibited relatively excellent electrochemical performance, but it still has a certain gap with the TEC of SDC electrolyte. To surmount this limitation, SDC was introduced to the NBCFM cathode to improve the thermal suitability of the cell’s module and extend the triple phase boundary (TPB) length.

### 3.2. Chemical Compatibility and Thermal Expansion Matching

**Figure 2:**
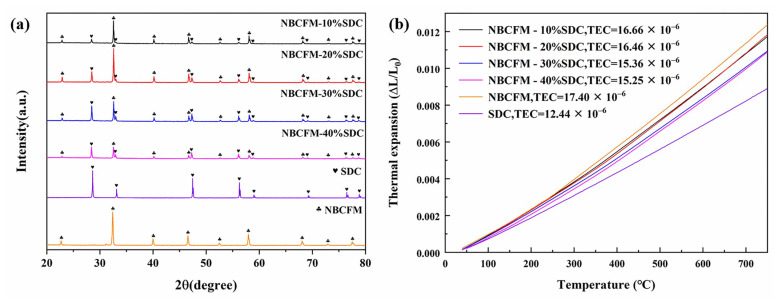
(**a**) The XRD profiles of the SDC, NBCFM, and NBCFM-*x*SDC powders after calcination at 1000 °C; (**b**) thermal expansion curves of SDC, NBCFM, and composite cathodes.

The NBCFM and SDC powers were mixed and co-calcined at 1000 °C for 4 h to identify the chemical compatibility. The phase composition of NBCFM-*x*SDC was analyzed by XRD. Figure 2a shows the XRD patterns of NBCFM, SDC, and NBCFM-*x*SDC. All diffraction peaks of the composite cathodes could be indexed well based on triple perovskite NBCFM and fluorite SDC phases, suggesting that no observable reaction is detected in the binary-mixed NBCFM-SDC systems.

Figure 2b shows the thermal expansion curves of NBCFM-*x*SDC composites and SDC between 40 and 750 °C. The TEC values are inserted in the figure. As expected, the TEC of the composite cathode decreases with the increasing of SDC content because of the lower TEC of SDC. The TEC values of NBCFM-30%SDC and NBCFM-40%SDC composite cathodes are 15.36 and 15.25 × 10^−6^ K^−1^, respectively, which are significantly lower than the TEC of NBCFM cathode. Despite the fact that the TEC value of NBCFM-*x*SDC is higher than that of SDC (~12.44 × 10^−6^ K^−1^), it is much lower than those of cobalt-containing cathodes, such as PrBa_0.5_Sr_0.5_Co_2−x_Fe_x_O_5+δ_ (*x* = 0.5, 1.0, 1.5, TEC = 19.2~21.3 × 10^−6^ K^−1^) [30] and PrBaCo_1.9_Ni_0.1_O_5+δ_-*x*SDC (*x* = 0, 10, 20, 30, 40 wt.%, TEC = 17.5~22.63 × 10^−6^ K^−1^) [31]. The decreases in TEC values can enhance the interface compatibility between the cathode and electrolyte and consequently improve the long-term stability.

### 3.3. Electrical Conductivity

**Figure 3:**
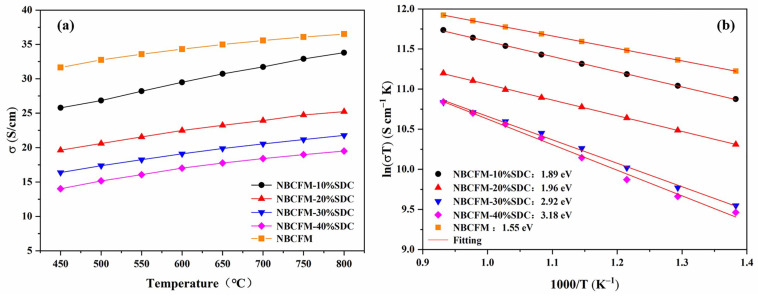
(**a**) Temperature dependence and (**b**) Arrhenius plots of the electrical conductivity for NBCFM and NBCFM-*x*SDC.

Figure 3a shows the temperature dependence of the electrical conductivity of NBCFM-*x*SDC composite cathodes in 450–800 °C. The Arrhenius plots of the total conductivity for NBCFM-*x*SDC are presented in Figure 3b, which shows a linear relationship between ln(σT) and 1000/T. Because the mobility of electrons is much faster than oxygen ions, the electrical conductivity of NBCFM and NBCFM-*x*SDC is mainly dominated by electronic conductivity. The conductivity of NBCFM increases with the rising temperature and reaches the maximum (36.51 S·cm^−1^) at 800 °C, indicating a typical semiconductor behavior. This behavior can be ascribed to electron hopping under thermal activation. For the perovskite materials containing cobalt, the electron conduction is mainly generated by electron hopping between Co^3+^/Co^4+^. The increase in temperature provides more energy for electron hopping and promotes carrier migration. However, the electrical conductivity of NBCFM-*x*SDC decreases with the increase of SDC content. In fluorite SDC, doping Ce^4+^ by Sm^3+^ leads to the formation of oxygen vacancies. The ions migrate rapidly via oxygen vacancies, meaning that the SDC exhibits high ionic conductivity. However, the oxygen vacancies take a disadvantage to the migration of carriers and disturb the (Co, Fe, Mn)-O-(Co, Fe, Mn) periodic potential, resulting in the localization of carriers and the decrease of electronic conductivity [32]. On the other hand, the conductivity of all NBCFM-*x*SDC is lower than that of NBCFM, related to the obstruction of contact between NBCFM particles by SDC particles. The path length of electron migration increases in the composite cathode, leading to a decrease in electron conductivity.

The activation energy for the migration of carrier (*E_a_*) can be calculated by Arrhenius Equation (1):

(1) $$\sigma =\left(\frac{A}{T}\right)\exp\left(\frac{-E_{a}}{kT}\right)$$

where *A* is the pre-exponential factor and *k* is the Boltzmann’s constant. The *E_a_* values of NBCFM-*x*SDC are shown in Figure 3b. The activation energy increases with the increase of SDC content. With the addition of SDC, the conductivity activation energy of the NBCFM-*x*SDC cathodes increases from 1.55 to 3.18 eV. The conductivity activation energy of NBCFM-40%SDC is 1.7 times higher than that of NBCFM.

### 3.4. Oxygen Surface Exchange Process

The ORR catalytic activity of mixed ionic–electronic conductors depends on two oxygen diffusion kinetic processes: one is the surface oxygen exchange process, and the other is the oxygen ion diffusion process. Oxygen diffusion kinetics works like a key to the catalytic activity of ORR and the oxygen ionic conductivity. The electrical conductivity relaxation (ECR) technique was utilized to characterize the oxygen diffusion behavior. For the samples with dimensions 2l_1_ × 2l_2_ × 2l_3_ (6 mm × 2 mm × 64 mm), the length is far larger than the width and height. The relationship between relative conductivity *f*(*t*) and time (*t*) is given by Equation (2) [33]:

(2) $$1-f\left(t\right)=1-\frac{\sigma_{t-}\sigma_{0}}{\sigma_{\infty }-\sigma_{0}}\;=\sum_{i=1}^{\infty }\sum_{n=1}^{\infty }\sum_{m=1}^{\infty }\frac{2C_{1}^{2}\exp\left(-\frac{\beta_{i}^{2}D_{0}t}{l_{1}^{2}}\right)}{\beta_{i}^{2}\left(\beta_{i}^{2}+C_{1}^{2}+C_{1}\right)}\times \frac{2C_{2}^{2}\exp\left(-\frac{\gamma_{n}^{2}D_{0}t}{l_{2}^{2}}\right)}{\gamma_{n}^{2}\left(\gamma_{n}^{2}+C_{2}^{2}+C_{2}\right)}\times \frac{2C_{3}^{2}\exp\left(-\frac{\delta_{m}^{2}D_{0}t}{l_{3}^{2}}\right)}{\delta_{m}^{2}\left(\delta_{m}^{2}+C_{3}^{2}+C_{3}\right)},$$

where *σ_t_*, *σ*_0_, and *σ_∞_* represent the time-dependent, initial, and final conductivities, respectively. *C_x_* is defined as the ratio of the half thickness to the characteristic length of the sample, *l_d_*.

(3) $$C_{x}=\frac{l_{x}}{l_{d}},$$

(4) $$l_{d}=\frac{D_{0}}{k_{chem}},$$

The coefficients *β_i_*, *γ_n_*, and *δ_m_* are the nonzero root of the transcendental equation:

(5) $$\beta_{i}\tan\beta_{i}=C_{1},\;\;\gamma_{n}\tan\gamma_{n}=C_{2},\;\;\delta_{m}\tan\delta_{m}=C_{3},$$

The oxygen transport of the sample is entirely controlled by the surface exchange process when *C_x_* in Equation (3) tends to zero. This indicates that the surface exchange process of the sample is much slower than the diffusion in the bulk phase. When the dimensions of the sample in one dimension (*l*_3_) are much longer than the other dimensions (*l*_1_, *l*_2_), the oxygen exchange process is mainly considered in the direction of the smaller dimension. In the smaller dimensional direction, the diffusion process can be ignored, and the oxygen exchange process is dominant since the diffusion path of oxygen is very short. At this time, Equation (2) can be simplified to an analytic function containing only the exchange coefficients:

(6) $$1-\frac{\sigma_{t-}\sigma_{0}}{\sigma_{\infty }-\sigma_{0}}=\exp\left(\frac{-k_{chem}t}{{\left(l_{1}^{-1}+l_{2}^{-1}\right)}^{-1}}\right),$$

**Figure 4:**
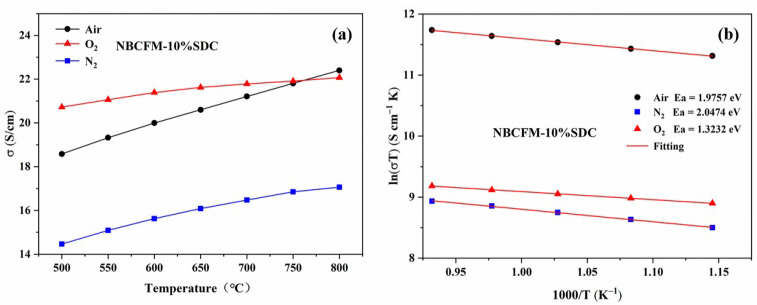
(**a**) Conductivity and (**b**) Arrhenius plots of the electrical conductivity for NBCFM-10%SDC at different temperatures (500–800 °C) in different atmospheres.

The changing of the external atmosphere has a specific effect on the concentration of internal oxygen vacancies for mixed conductor oxide. This change in the oxygen vacancy concentration will affect its electrical properties, which is the basis for the electrical conductivity relaxation test. Figure 4 shows the conductivity test results of NBCFM-10%SDC under nitrogen, air, and oxygen atmospheres. In the whole test temperature range, the conductivity in the nitrogen atmosphere is significantly lower than that in air and oxygen atmospheres. The defect response that occurs during the test can be described by Equation (7).

(7) $$O_{O}^{\times }+2h^{\cdot }⟺\frac{1}{2}O_{2}\left(g\right)↑+V_{O}^{\cdot \cdot },$$

The ambient oxygen partial pressures are different in the three test atmospheres. To balance the lower oxygen partial pressure in a nitrogen atmosphere, the reaction moves to the right. The surface oxygen escapes from the lattice with a decrease in electron holes and an increase in oxygen vacancies, resulting in the ionic conductivity increasing and the electronic conductivity decreasing. Similarly, the reaction moves to the left under the oxygen atmosphere. The increase of electron holes and the reduction of oxygen vacancies increase the electronic conductivity and decrease the ionic conductivity. Although the contribution of ionic conductivity increases in the medium and high temperature stages, the electronic conductance still dominates. The apparent total conductivity is *σ*(O_2_) > *σ*(Air) > *σ*(N_2_). Therefore, it can be seen that changes in the external environment of NBCFM-*x*SDC can certainly have some effect on its internal oxygen defect concentration.

**Figure 5:**
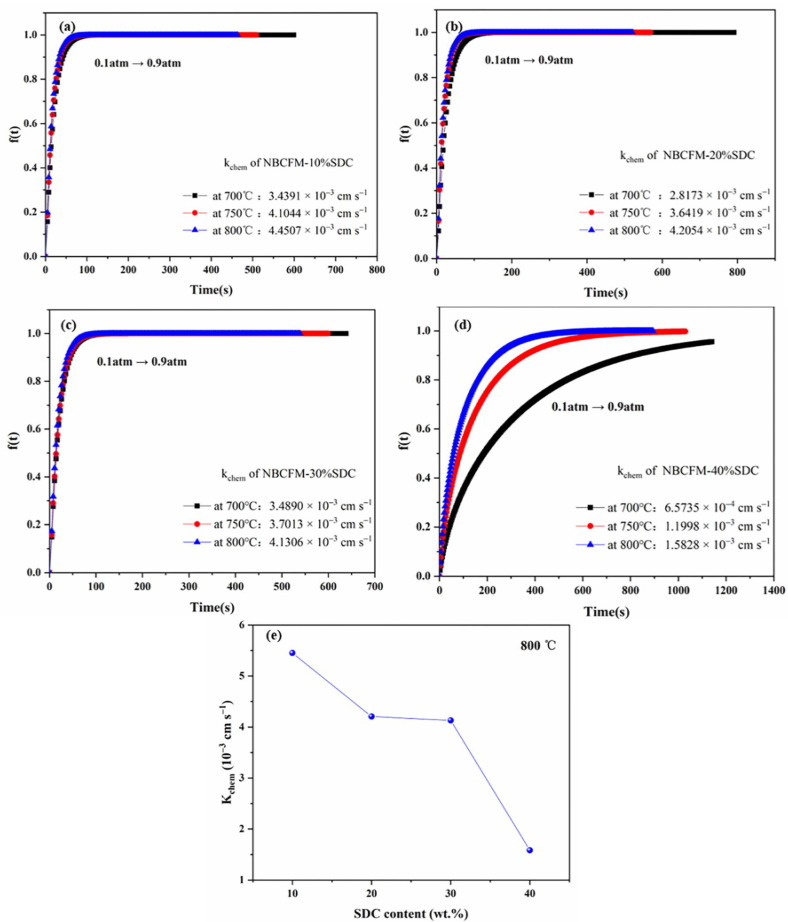
Conductivity relaxation plots of (**a**) NBCFM-10%SDC, (**b**) NBCFM-20%SDC, (**c**) NBCFM-30%SDC and (**d**) NBCFM-40%SDC at 700 °C, 750 °C and 800 °C; variation of (**e**) *k_chem_* of NBCFM-*x*SDC with SDC content.

In order to study the effect of SDC content on the surface kinetics process, the surface exchange coefficient (*k_chem_*) is measured by the ECR method. Figure 5 shows the conductivity relaxation plots for NBCFM-*x*SDC in the temperature range of 700–800 °C. It can be seen that the trend of the curve changes from steep to flat as the oxygen partial pressure changes from 0.1 to 0.9 atm. This dynamic equilibrium process reflects the change of the oxygen vacancy concentration. The equilibrium time gradually decreases with the increase of testing temperature because ions gain more energy to move by thermal excitation. The calculated *k_chem_* values of NBCFM-*x*SDC are presented in Figure 5, where the decrease of the surface exchange coefficient with increasing SDC content can be observed. NBCFM-10%SDC has a maximum *k_chem_* of 4.4507 × 10^−3^ cm s^−1^ at 800 °C, which is higher than that of other composite cathodes, as given in Figure 5e. The surface exchange process usually includes oxygen absorption, dissociation, and reduction at the surface, and oxygen ions move into the cathode. As the ionic conductor content increases, the electron transport distance becomes longer and the concentration of surface electron reduces, resulting in a slower oxygen reduction process on the surface. In addition, the ionic potentials of samarium (Sm^3+^~1.410) and cerium (Ce^4+^~1.608) ions are stronger than those of neodymium (Nd^3+^~1.382), Ba^2+^~1.126, Co^2+^~1.377 (LS), Fe^2+^~1.390 (LS), or Mn^2+^~1.343 (LS) [34] ions. Elements with higher ionic potential are more strongly bound to oxygen; thus, in NBCFM-*x*SDC with higher SDC content, oxygen escape is more difficult, and a lower surface exchange coefficient *k_chem_* is exhibited. In addition, more energy is required for the ions to jump between the two-phase interfaces in the composite cathode. As the second phase content increases, the ion transport distance becomes longer and the concentration of surface oxygen reduces, leading to a slower overall oxygen surface exchange process.

### 3.5. Electrochemical Performance and the Microstructure

The ORR rate of MIEC materials is affected by the concentration of oxygen vacancies and microstructure. A high vacancy concentration provides more sites for the oxygen incorporation, and the microstructure reflects the length of the TPB. To obtain further information on the ORR catalytic activity of NBCFM-*x*SDC, the Electrochemical Impedance Spectroscopy (EIS) is fitted by the Zview software using the equivalent circuit L-R_Ω_ (R_P1_-CPE_R1_)-(R_P2_-CPE_R2_) [35]. L is the high frequency inductance, which is caused by the cathode system and the lead wire. R_Ω_ is related to the ohmic resistance of the electrolyte. To clearly show the differences in the cathodic polarization behavior, the ohmic resistances measured in this study are normalized to 0 when the electronic conduction in the electrolyte is negligible. R_P1_-CPE_R1_ and R_P2_-CPE_R2_ correspond to a high frequency arc and a low frequency arc, respectively. The higher frequency R_P1_ and the lower frequency R_P2_ are related to the processes of charge transfer and oxygen diffusion, respectively [36,37]. CPE_Ri_ is a constant phase component. The total polarization resistance R_P_ is the sum of R_P1_ and R_P2_. The area specific resistance (ASR) can be determined by the following relationship: ASR = R_P_ × S/2 [38,39], where S is the electrode surface area.

**Figure 6:**
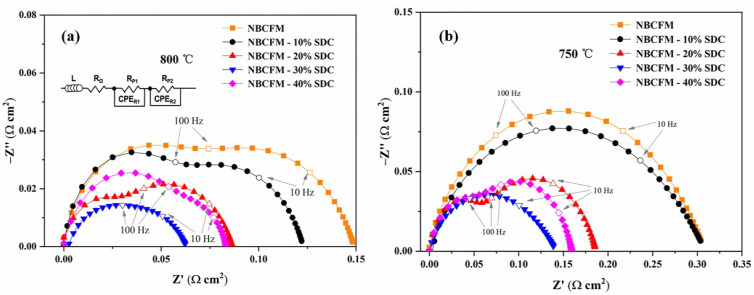
Impedance spectra of the NBCFM and composite electrodes at (**a**) 800 °C and (**b**) 750 °C.

The impedance spectra for NBCFM-*x*SDC are shown in Figure 6. The ASR values significantly depend on the relative mass ratio of SDC and NBCFM (Table 2). The NBCFM-30%SDC sample exhibits the lowest ASR values of 0.06575 Ω cm^2^ at 800 °C. Under SOFC operating conditions, the highly ionic conductive SDC is known to have a good ability to store, release, and transport oxygen. The appropriate amount of SDC can greatly improve the oxygen catalytic activity. The ASR value of NBCFM-30%SDC is only 41% of that of NBCFM. The better performance for NBCFM-*x*SDC is believed to be due to the good adhesion between the cathode and electrolyte and the faster charge transfer. By introducing SDC as a second phase, the ion diffusion path in the cathodes is shortened, and the TPB areas of the composite electrode are extended. As a result, the distance between oxygen vacancies is reduced and the ionic conductivity is enhanced, thereby increasing the oxygen ion transport capacity. The thermal expansion mismatch of NBCFM-*x*SDC is also weakened with the increase of SDC content. In conclusion, the improvement in thermal expansion properties and the increase in ionic conductivity enhance the electrochemical properties of NBCFM-*x*SDC. The ASR value of NBCFM-30%SDC is lower than that of Gd_0.8_Sr_0.2_CoO_3−δ_-20 Sm_0.1_Ce_0.9_O_1.95_ (0.16 Ω cm^2^ at 750 °C) [38] and YBa_0.5_Sr_0.5_Co_1.4_Cu_0.6_O_5+δ_-30%SDC composite cathode (0.034 Ω cm^2^ at 800 °C) [40]. However, the R_P_ increases when the SDC content is higher than 30 wt.%. The increase of R_P_ can be ascribed to the enlarged electronic transport path and slow surface exchange kinetics. As the SDC content increases, the connectivity of NBCFM particle decreases. The path of electronic transport may be obstructed and the rate of electrochemical reaction decrease, resulting in an increase of R_P_.

**Figure 7:**
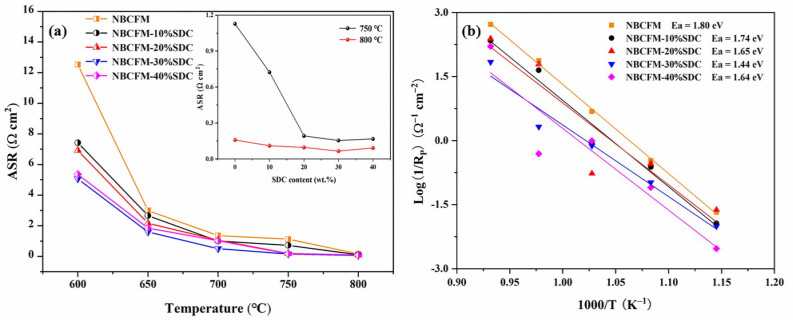
(**a**) ASR values for NBCFM-*x*SDC composite cathodes at 600–800 °C in air; inset shows dependence of ASR for NBCFM-*x*SDC composite cathodes with different SDC contents at 750 °C and 800 °C; (**b**) Arrhenius plots of the polarization resistance of the NBCFM-*x*SDC composite cathode materials.

**Figure 8:**
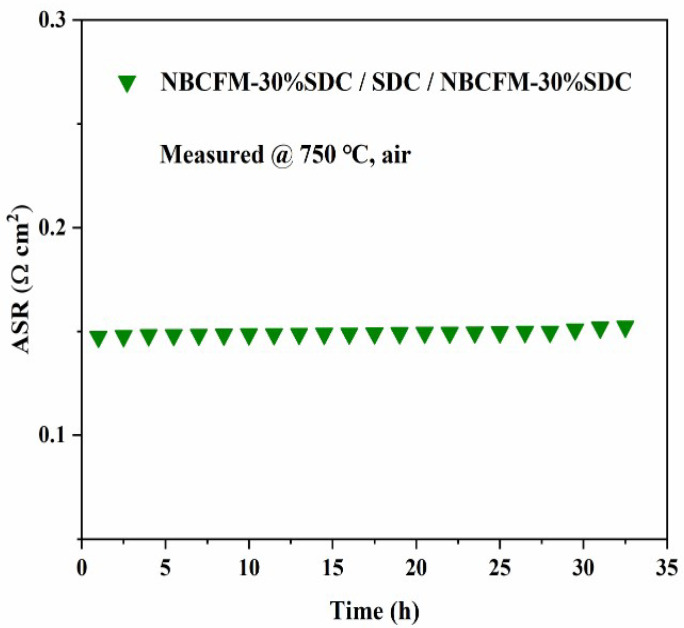
Short-term stability of a symmetric cell at 750 °C in air.

Figure 7a displays the ASR values of NBCFM-*x*SDC with the temperature, while the ASR values of NBCFM-*x*SDC as a function of the SDC contents at 750 and 800 °C are also shown in the inset of Figure 7. This shows clearly that the content of SDC has a significant effect on ASR. The ASR values of NBCFM-*x*SDC composite cathodes decrease at first and then begin to increase with increasing SDC content. Figure 7b shows the Arrhenius plots of R_P_ values as a function of temperature, and the activation energy is calculated from the slope of the curves. It can be observed that R_P_ of NBCFM-*x*SDC has a linear behavior with temperature. All the composites have a lower activation energy than NBCFM, suggesting that the incorporation of SDC into NBCFM can effectively lower the barrier for cathode reaction and improve the electrochemical performance of the cells. The result also shows that the composite incorporated with 30 wt.% SDC exhibits optimal performance in the present experiment. In order to examine NBCFM-30%SDC stability in operating conditions, the ASRs of a symmetric cell were periodically measured at 750 °C in air. As can be seen in Figure 8, the cell shows a fairly stable performance without any detectable degradation for 33 h.

**Figure 9:**
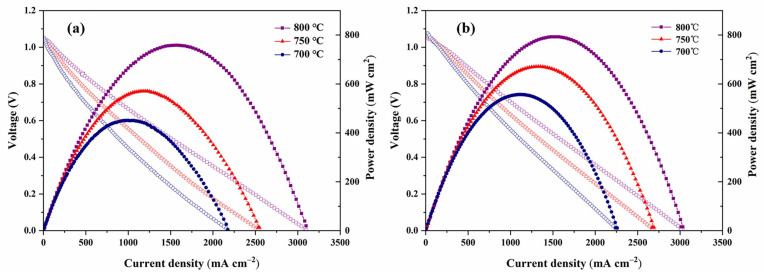
I-V and I-P curves of single cells at different temperatures (700–800 °C) for cathode (**a**) NBCFM and (**b**) NBCFM-30%SDC.

NBCFM-30%SDC composite oxide is evaluated as a cathode material due to its relatively lower TEC values and polarization resistance. The anode-supported single cell with the configuration of Ni-YSZ/YSZ/GDC/NBCFM-30%SDC (or NBCFM) is constructed, and the cell performance is shown in Figure 9. The power density increases with the increase of operating temperature. The peak power densities (PPDs) of the cell using NBCFM as a cathode were 451.03 mW cm^−2^, 570.34 mW cm^−2^, and 757.79 mW cm^−2^ at 700 °C, 750 °C, and 800 °C, respectively. Furthermore, the PPDs of the single cells using NBCFM-30%SDC as a cathode were 556.76 mW cm^−2^, 670.75 mW cm^−2^, and 792.56 mW cm^−2^ at 700 °C, 750 °C, and 800 °C, respectively. The NBCFM-30%SDC cathode showed better cell performance, which is consistent with the ASR results. It is believed that the SDC-matched TEC and low ASR value of NBCFM-30%SDC make a great contribution to high power density.

**Figure 10:**
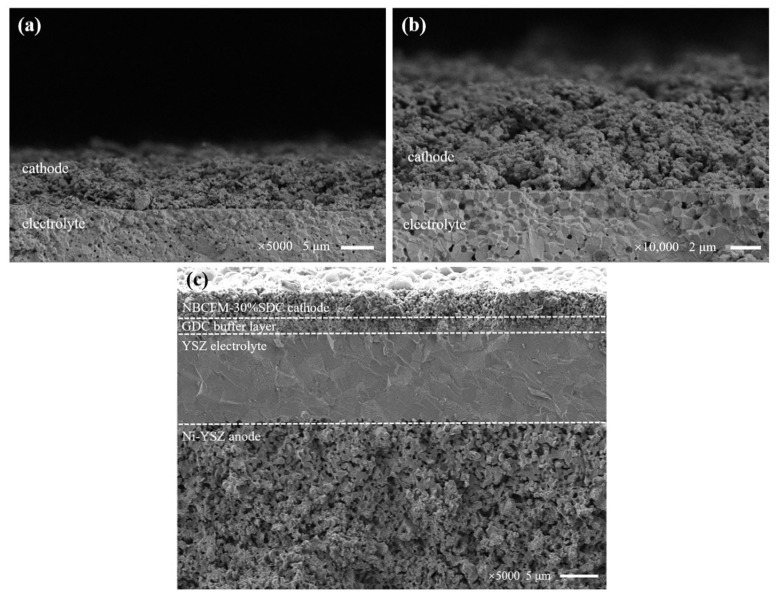
SEM micrograph interface-view for (**a**,**b**) NBCFM-30%SDC/SDC cathodes, and (**c**) Ni-YSZ/YSZ/GDC/NBCFM-30%SDC single cell after cell test.

The SEM images of NBCFM-30%SDC are shown in Figure 10. The coarse and fine particles can be observed in the cross-sectional images of the composite cathodes, where the smaller particles are in the SDC phase. It can be seen that the good contact between the porous NBCFM-30%SDC cathodes and the dense SDC electrolyte layers ensures the low cathode/electrolyte interface resistances in the NBCFM-30%SDC/SDC/NBCFM-30%SDC symmetrical cell. The uniform and continuous distribution of ionic conductors leads to better oxygen ions diffusion for the ORR, resulting in the good electrochemical performance of NBCFM-30%SDC cells.

## 4. Conclusions

In this work, triple perovskite Nd_1.5_Ba_1.5_CoFeMnO_9−δ_ and a series of NBCFM-*x*SDC composite cathodes were successfully prepared, and their feasibility as SOFC cathodes was evaluated. The NBCFM with high oxygen vacancies had low polarization impedance, exhibiting good electrochemical catalytic performance. The power density of a single cell using NBCFM as a cathode was 757.79 mW cm^−2^ at 800 °C. NBCFM-*x*SDC had good chemical stability because NBCFM oxide had good chemical compatibility with SDC. Electrical conductivities and TECs decreased monotonously with increasing SDC content. The TEC decreased from 17.40 × 10^−6^ K^−1^ for NBCFM to 15.25 × 10^−6^ K^−1^ for NBCFM-40%SDC. However, the conductivity values of the NBCFM-*x*SDC cathode are not extraordinary and still need to be improved. The ECR results showed that the oxygen surface exchange rate of NBCFM-*x*SDC slowed down with increasing SDC content, and its surface oxygen exchange coefficients were within the range of 10^−4^ to 10^−3^ cm s^−1^ at the tested temperature. Among composite cathodes, the NBCFM-30%SDC exhibited the lowest polarization resistance. The maximum power density of NBCFM-30%SDC based on an anode-supported single cell achieved 792.56 mW cm^−2^ at 800 °C. The result indicates that NBCFM-30%SDC is a promising cathode material for SOFC application.

## Figures and Tables

**Table 1:** The lattice parameters and the unit cell volume.

		NBCFM	SDC
**Phase structure**		Tetragonal	Cubic
**Space group**		P4/mmm	Fm–3m
**Lattice parameter**	a (Å)	3.8855	5.411
	b (Å)	3.8855	5.411
	c (Å)	11.6897	5.411
**Unit cell volume (Å^3^)**		176.4807	158.428

**Table 2:** The fitted results of ASR for NBCFM-*x*SDC composite cathodes measured at 800 °C in air.

Samples	R_P1_ (Ω cm^2^)	R_P2_ (Ω cm^2^)	ASR (Ω cm^2^)
NBCFM	0.1073	0.0514	0.1587
NBCFM-10%SDC	0.0615	0.0487	0.1102
NBCFM-20%SDC	0.0323	0.0640	0.0963
NBCFM-30%SDC	0.0234	0.0422	0.0656
NBCFM-40%SDC	0.0667	0.0252	0.0919

## Data Availability

All data that support the findings of this study are included within the article.
